# Neuropilins are multifunctional coreceptors involved in tumor initiation, growth, metastasis and immunity

**DOI:** 10.18632/oncotarget.626

**Published:** 2012-09-03

**Authors:** Gérald J. Prud'homme, Yelena Glinka

**Affiliations:** ^1^ Keenan Research Centre in the Li Ka Shing Knowledge Institute of St. Michael's Hospital, ON, Canada; ^2^ Department of Laboratory Medicine, St. Michael's Hospital, Toronto, ON, Canada; ^3^ Department of Laboratory Medicine and Pathobiology, University of Toronto, Toronto, ON, Canada

**Keywords:** angiogenesis, cancer stem cell, growth factor, neuropilin, semaphorin, TGF-beta, VEGF

## Abstract

The neuropilins (Nrps) are multifunctional proteins involved in development, immunity and cancer. Neuropilin-1 (Nrp1), or its homologue neuropilin-2 (Nrp2), are coreceptors that enhance responses to several growth factors (GFs) and other mediators. Nrps are coreceptors for the class 3 semaphorins (SEMA3), involved in axonal guidance, and several members of the vascular endothelial growth factor (VEGF) family. However, recent findings reveal they have a much broader spectrum of activity. They bind transforming growth factor β1 (TGF-β1) and its receptors, hepatocyte growth factor (HGF) and its receptor (cMet), platelet derived growth factor (PDGF) and its receptors, fibroblast growth factors (FGFs), and integrins. Nrps also promote Hedgehog signaling. These ligands and pathways are all relevant to angiogenesis and wound healing. In the immune system, the Nrps are expressed primarily by dendritic cells (DCs) and regulatory T cells (Tregs), and exert mainly inhibitory effects. In cancer, Nrps have been linked to a poor prognosis, which is consistent with their numerous interactions with ligands and receptors that promote tumor progression. We hypothesize that Nrps boost responses by capturing ligands, regulating GF receptor expression, endocytosis and recycling, and possibly also by signaling independently. Importantly, they promote epithelial-mesenchymal transition (EMT), and the survival of cancer stem cells. The recent finding that Nrps bind and internalize cell-penetrating peptides (CPPs) with arginine/lysine-rich C-terminal motifs (C-end rule; e.g., RXXR) is of interest. These CPPs can be coupled to large drugs for cancer therapy. Almost all studies have been preclinical, but findings suggest Nrps are excellent targets for anti-cancer drug development.

## INTRODUCTION

The neuropilins (Nrps) are multifunctional single-pass transmembrane proteins that play an important role in development, immunity and cancer [1-14]. Neuropilin-1 (Nrp1), or its homologue neuropilin-2 (Nrp2), are coreceptors that enhance responses to several growth factors and other mediators under physiological and pathological conditions. They are expressed by endothelial cells, several other normal cell types, and often by malignant tumor cells [5, 14-16]. Nrp1 and Nrp2 have 44% homology and share many structural and biological properties [1, 2, 9-14]. Nrps are usually expressed as homodimers, but Nrp1/Nrp2 heterodimers also occur [17]. Nrp1 (also denoted CD304 or BDCA-4) was first identified as a receptor for the class 3 semaphorins (SEMA3) [18-26], which are involved in axonal guidance in embryonic development. In this function, Nrp1 acts as a coreceptor for SEMA3 family members and promotes their interaction with plexins. Subsequently, the Nrps were identified as coreceptors for several members of the vascular endothelial growth factor (VEGF) family [27-32]. Nrp1 was found to interact with VEGF-A_165_ (and other VEGFs) and the receptor tyrosine kinase (RTK) VEGFR2, and to enhance signaling through this pathway and promote angiogenesis. Heparin markedly increases the affinity of VEGF for Nrp1, and appears to contribute to the formation of a complex incorporating VEGF, Nrp1 and VEGFR2 [1, 2, 28]. Nrp2 has different (but overlapping) binding preferences for VEGF family members, and is a coreceptor for VEGFR3 that is involved in lymphatic endothelial cell function [32]. In view of this, the current concept is that the Nrps are coreceptors for SEMA3 and VEGF family members.

**Figure 1:**
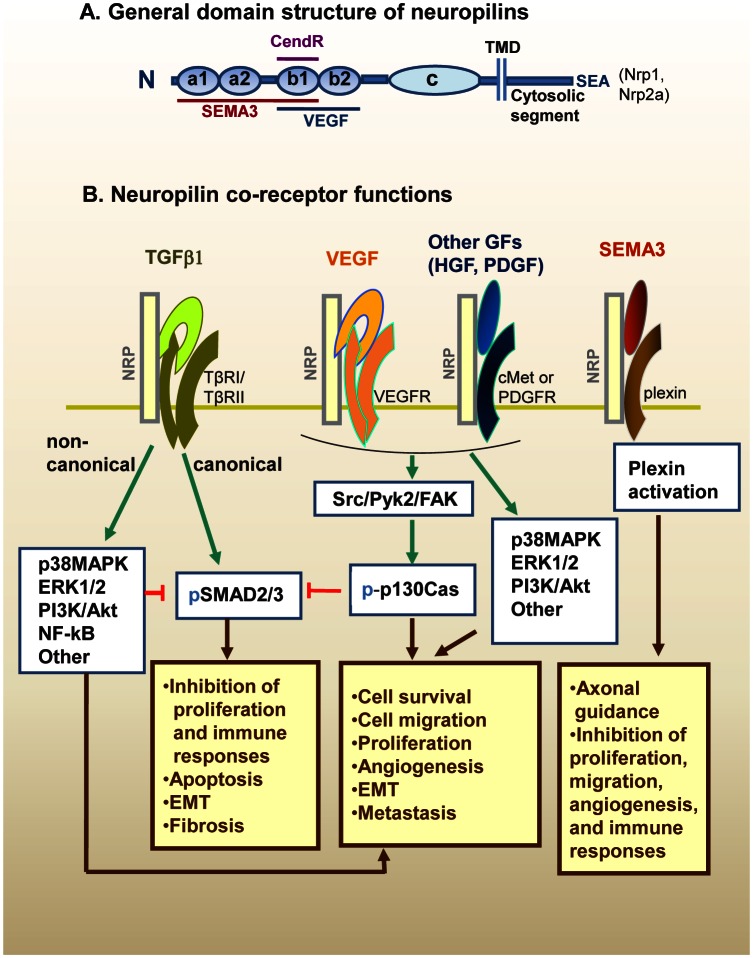
Neuropilin (Nrp) structure and hypothetical model of interaction with multiple growth factors A. The general domain structure of Nrp1 and Nrp2 is shown. There are five extracellular domains, a single-pass TMD domain, and a short cytosolic tail lacking tyrosine kinase activity. Nrp1 and Nrp2a (but not Nrp2b) have a C-terminal SEA-sequence motif that binds to synectin. There are also splice variants of the Nrps (not shown), including soluble forms lacking the c domain, TMD and cytoplasmic segments. SEMA3s bind to the a1/a2/b1 segment, and VEGFs binds to b1/b2. The binding sites of other GFs are not well characterized. CendR peptides bind to the b1 domain (see text). The c domain contributes to receptor dimerization. B. The Nrps bind ligands of at least five major types of soluble mediators, as well as their signaling receptors. The figure presents a hypothetical model of how these signaling pathways may interact. This includes TGF-β1, VEGF family, HGF, PDGF-BB, and the SEMA3 family. See Table 1 for a list of ligands and references. Except for SEMA3s, the Nrps are not essential for receptor signaling but they enhance the response. The GF signaling pathways intersect extensively with numerous potential outcomes. TGF-β exerts antiproliferative and immunosuppressive effects through canonical (Smad2/3) signaling. However, TGF-β noncanonical signaling (or other GF pathways) can inhibit Smad2/3 signaling. Notably, p130Cas is phosphorylated in response to Nrp-binding GFs, and can block Smad2/3 signaling while favoring noncanonical signaling. SEMA3s interact with Nrps and plexins (the signaling receptors) to activate signaling pathways that regulate axonal guidance, as well as endothelial, immune and tumor cell responses, usually in an inhibitory way. Abbreviations: CendR; C-end rule peptides; cMet, hepatocyte growth factor receptor; EMT, epithelial-to-mesenchymal transition; FAK, focal adhesion kinase; GF, growth factor; HGF, hepatocyte growth factor; Nrp1, neuropilin-1; Nrp2, neuropilin-2; p-, phosphorylated form; p130Cas, Crk-associated substrate; PDGF, platelet-derived growth factor; PDGFR; PDGF receptor; Pyk2, proline-rich tyrosine kinase 2; SEMA3, class 3 semaphorin; TGF-β, transforming growth factor-β; TGF-βRI, TGF-β receptor type 1 (also denoted ALK5); TGF-βRII, TGF-β receptor type 2; TMD; transmembrane domain; VEGF, vascular endothelial growth factor; VEGFR, VEGF receptor.

Interestingly, the activation of VEGF receptors by VEGF can occur in the absence of Nrps [13], but Nrp1 mediates endothelial cell migration and is essential in angiogenesis as shown in knockout mice. This could occur because Nrps boost responses to VEGF, and a small decrease in VEGF availability can have major consequences as demonstrated by the embryonic death of mice lacking even one allele of the VEGF gene. However, alternatively, this might occur because Nrp1 also interacts with other key receptors involved in angiogenesis. Indeed, new findings show that the Nrps have a much broader spectrum of ligands than initially recognized [33-63], as outlined in Table 1 and Figure 1A,B. Nrp1 can bind transforming growth factor β1 (TGF-β1) and its receptors [33-36], hepatocyte growth factor (HGF) and its receptor (cMet) [37-39, 45], platelet derived growth factor (PDGF) and its receptors [40-45], and some fibroblast growth factors (FGFs) [37]. However, Nrp1 had no effect on the response of human umbilical vein endothelial cells (HUVEC) to FGF-2 [64], and the relevance of interactions with FGFs remains unclear. Nrp1 also interacts with integrins [46-48], numerous synthetic or natural cell-penetrating peptides (CPPs) [53-57], and other molecules. Nrps contribute to cell adhesion in embryonic cells and some other cell types [65]. A caveat is that the importance of other Nrp ligands (non-VEGF) in the context of angiogenesis has not been established, and a considerable amount of work will be required to elucidate their role. In addition, the Nrps are involved in the regulation of Hedgehog (Hh) signaling [66, 67], and the survival and self-renewal of cancer stem cells [68-70]. Nrp1 also binds to itself [37], which may be relevant to some cell-cell interactions. The molecular features responsible for this remarkable variety of interactions are for the most part unknown, but crystallographic studies and other observations have provided valuable information as outlined below.

**Table 1:** Neuropilin ligands

Ligand	Nrp1	Nrp2	References
			
VEGF-A121	+		9-14,27-32
VEGF-A145		+	
VEGF-A165	+	+	
VEGF-B167	+		
VEGF-C	+	+	
VEGF-D	+	+	
VEGF-E	+		
PlGF-2	+	+	
VEGFR	+(R1/R2)	+(R1/R2/R3)	
			
Heparin	+		28, 30, 107
			
SEMA3A	+		18-26
SEMA3B,C,D,F	+	+	
SEMA3G		+	
Plexin-A1 to A4; D1	+	+	
			
TGF-β1 and LAP	+	+	33-36
TβRI and TβRII	+	+	
			
HGF and cMet	+	+	37-39, 45
			
PDGF and PDGFR	+		40-45
			
FGF-1, 2, 4, 7*	+		37
FGF receptor-1*	+		
			
Integrins	+	+	46-48, 134
(α5β1; αvβ3; other)			
			
Fibronectin	+		134
			
Galectin-1	+		49
			
L1-CAM	+	+	50, 51
			
Glut-1	+		52
			
Peptides (CendR:	+	+	53-62
^R/^_K_XX^R/^_K_; others)			
			
Neurotrophin R	+		63

+ the ligand binds to neuropilin; R, receptor; PlGF-2, placenta growth factor 2; for other abbreviations see the legend to Figure 1.

* Nrp1 did not to alter the response of HUVEC to FGF-2 [64].

The importance of Nrps in angiogenesis and axonal guidance is firmly established, as noted above. However, their role in immunity, cancer and other processes is not as clear. In this review, we will focus mainly on immune aspects and cancer, especially as related to non-classical Nrp ligands such as TGF-β and HGF. We will also examine the potential of Nrps as targets for cancer therapy.

## PROPERTIES OF THE NRPS

### Cells expressing Nrp1 and Nrp2 in normal tissues and tumors

Nrp1 and/or Nrp2 expression has been reported in a wide variety of cells including endothelial cells, neurons, pancreatic islet cells, hepatocytes, melanocytes and osteoblasts [6, 14-16]. Nrp expression has been studied both *in vitro* and *in vivo*, and in most cases confirmed *in vivo*. In addition, expression occurs in some epithelial cells of several organs (e.g., skin, breast, prostate, GI tract, lung, kidney and bladder), as recently reviewed [14]. The endothelial cells of arteries express primarily Nrp1, whereas the endothelial cells of veins and lymphatics express predominantly Nrp2. In the immune system, Nrp1 is expressed by thymocytes [71-74], plasmacytoid dendritic cells (pDCs) [75-76], and regulatory T cells (Tr or Treg cells) [4, 33, 77-79]. In mice, Nrp1 is expressed by the majority of resting or activated Treg cells [4], while in humans it is poorly expressed by resting Treg cells but induced in a subpopulation of activated Treg cells [80, 81]. Nrp1 is also expressed by recent thymic emigrant IL-17-producing invariant NKT cells in lymphoid organs, and is a useful marker for these cells [82].

Some studies have shown discordant results regarding the expression of Nrp1 and Nrp2 in normal tissues and tumors, which probably reflects technical differences. Jubb et al. [15, 16] addressed this question by staining with antibodies that were strictly validated for immunohistochemical analysis, and they also employed in situ hybridization. In the case of tumors, they observed that the vasculature is positive for Nrp1 [15] and Nrp2 [16] in the vast majority of cases. However, Nrp expression by tumor cells varied considerably from one tumor type to another. For example, Nrp1 positivity by tumor cells was recorded in 6% of primary and 14% of metastatic breast cancers, and 36% of primary and 50% of metastatic non-small-cell lung carcinomas (NSCLCs) [15]. The frequency of tumors with Nrp2+ tumor cells was comparable to Nrp1 in breast cancer and NSCLC [16]. In contrast, 85% of melanomas had Nrp2+ tumor cells. In positive tumors, the percent of Nrp+ tumor cells ranged from small to almost 100%. These marker profiles were determined by single Nrp1 or Nrp2 staining, and the frequency of positive tumors would likely be higher if analyzed by double staining. It should be noted that others have reported a much higher frequency of Nrp2 expression (~ 50%) by tumor cells in breast cancer [81]. The potential significance of Nrp expression in tumors is addressed in another section below.

### Structural features of Nrp1 and Nrp2 and coreceptor functions

The molecular features of Nrp1 and Nrp2 (Nrp2a and Nrp2b) have been recently reviewed [1, 2, 10-14], and only a brief outline is provided here. Nrp1 and Nrp2a are 130-140 kd type-1 membrane glycoproteins with 44% sequence homology, similar domain structure and an overlapping set of ligands (Fig. 1A). They have an extracellular portion consisting of five domains, a transmembrane segment, and a short cytoplasmic tail of ~44 amino acids (aa). The cytoplasmic segment has no known signaling motif, but in Nrp1 and Nrp2a there is a C-terminal SEA motif that interacts with the PDZ protein denoted neuropilin-1 interacting protein (NIP), GIPC or synectin [46, 84-87]. The extracellular domains consists of a1/a2, b1/b2 and c (MAM) domains. The a1/a2 domains (or CUB domains) consist of ~110 aa each and have homology of the C1s/C1r complement proteins. The b1/b2 domains are ~150 aa each, and have homology to the C1/C2 domains of coagulation factors V and VIII. The c domain is thought to be involved receptor dimerization, but other segments may also contribute, including the transmembrane domain [89]. Notably, a synthetic peptide derived from the transmembrane segment of Nrp1 disrupted its coreceptor functions and showed therapeutic benefit in a glioblastoma model [89, 90].

In addition to these classic forms of neuropilin (Nrp1 and Nrp2a), several isoforms produced as splice variants have been described. This includes soluble forms of both Nrp1 and Nrp2, lacking the cytoplasmic, transmembrane and c domains. There is also an alternative membrane form of Nrp2 (Nrp2b) that lacks the cytoplasmic SEA motif and does not bind synectin. Although Nrp1 and Nrp2 appear to exist mostly as homodimers recent studies with mutants, particularly dominant negative Nrp1 mutated forms, reveal that Nrp1/Nrp2 heterodimers also occur [17]. The biological significance of these various Nrp1 and Nrp2 splice variants and heterodimers is not well understood, but they appear to be expressed differently in tissues and are likely to also differ in function.

Although Nrp1 and Nrp2 both have affinity for VEGF and SEMA3, there are differences, as seen in Table 1. Thus, Nrp1 interacts preferentially with SEMA3A, whereas Nrp2 favors SEMA3F. With respect to the VEGF family, both Nrp1 and Nrp2 bind multiple members, but there is not a complete overlap in reactivity (Table 1). The significance of these different affinities for various mediators is unknown. As noted previously, a remarkable feature of the Nrps is their ability to interact with both the ligands and receptors of at least five unrelated families of mediators (Fig. 1B, Table 1). In these interactions, numerous studies have suggested that Nrps are chiefly modulators of signaling, with VEGF being the prototypical example [39], but how they achieve this function is largely unknown. We speculate that this results from the ability of the Nrps to capture the soluble ligands on the cell membrane and increase their availability for interaction with the signaling receptors. This might occur as part of multi-component signaling complexes that are stabilized by the Nrps. In accord with this hypothesis, there is evidence that Nrp1 knockdown in some cell types results in a dramatic decrease in VEGFR2 protein levels [69]. Furthermore, in the case of TGF-β we have shown that the Nrps have an important role in the activation of the latent cytokine, as discussed below.

An alternative hypothesis, not necessarily compatible with the previous one, is that Nrps contribute to endocytosis and/or routing of the receptor complex, which is some cases enhances signaling. For instance, there is evidence that clathrin-mediated endocytosis promotes TGF-β receptor signaling [91, 92]. It has been reported that Nrp1, through its cytoplasmic C-terminal motif (SEA), acts as a general adaptor by binding to the cytoplasmic PDZ protein synectin (GIPC) [46, 93]. Synectin, along with other proteins such as Dab2, links vesicles of the clathrin-dependent endosomal pathway with molecular motor myosin VI (Myo6), to promote endosomal trafficking [93, 94]. However, synectin does not appear essential for some key Nrp functions [13], and its role in this context remains to be clearly defined.

### Phenotype of Nrp knockout mice

The importance of Nrps in development had been demonstrated in knockout mice. Nrp1 knockout in mice is embryonic lethal at 10 to 12.5 days [95, 96]. The embryos die with several defects in cardiac and vascular development, as well as disorganization of the pathway and projection of nerve fibers. Conditional Nrp1 knockout limited to endothelial cells is also associated with cardiac and vascular defects [97]. The study of mice expressing a mutant Nrp1 capable of binding VEGF, but not SEMA3, revealed that SEMA3 signaling is not required for vascular development but is essential for normal axonal pathfinding by neurons of the central and peripheral nervous system [98]. Interestingly, these mice had heart defects, suggesting a role for both VEGF and SEMA3 in cardiac development. Nrp1 overexpression in transgenic mice is also lethal at ~ 12.5 days, and is associated with cardiac malformations, increased blood vessels and capillaries, and nerve fiber anomalies [99]. These findings clearly confirm the key roles of Nrp1 in cardiac, vascular and nervous system development. In contrast, Nrp2 knockout mice are viable, but have decreased numbers of lymphatics and capillaries, and defects of the central and peripheral nervous system [100]. The embryos of Nrp1 and Nrp2 double-knockout mice exhibit more severe anomalies and die earlier than Nrp1 single-knockout mice [101].

### Putative Nrp1 binding sites for its ligands

The SEMA3s appear to bind to the a1/a2 domains, whereas the VEGFs bind to the b1/b2 domains. Although it has been thought these binding sites overlap, based on competition [102] and mutation [103] studies, some recent studies suggest that they do not but this remains controversial. For instance, Pan et al. [104] found that an anti-Nrp1 antibody (anti-Nrp1^A^) that blocks SEMA3 binding does not block VEGF, whereas an antibody (anti-Nrp1^B^) that blocks VEGF does not block SEMA3. This is in accord with the study of Appleton et al. [105]. These authors analyzed crystal structures of Nrp1 and Nrp2 fragments alone or bound to antibodies that selectively block either SEMA3 or VEGF. The location of the antibody epitopes as well as *in vitro* experiments suggested that VEGF and SEMA3 did not directly compete for binding. The analysis of Nrp1 domain deletions or mutations by Gu et al. [103] showed that the a1/a2 domains bind SEMA3, while the b1/b2 domains bind VEGF. However, deletion of the b1 domain also reduced SEMA3 binding. Of note, mutating seven amino acids in the a1 domain of Nrp1 abrogated its capacity to bind SEMA3, but did not prevent binding to VEGF, VEGFR2 or Plexin A1.

Recent crystal structure studies of Nrp1, especially of the b1 and b2 domains, as well as other evidence, reveal probable sites of interaction with a number of unrelated ligands. Lee et al. [106] reported that the b1 domain has a cleft with negative charge, and suggested that the positively charged C-terminal tails of VEGF and SEMA3 bind in this location. Vander Kooi et al. [107] examined the crystal structure of the b1 and b2 domain, with bound Tuftsin (TKPR), a peptide mimetic of the exon-8 C-terminal motif of VEGF_165_ (KPRR). Tuftsin competes with VEGF_165_ for binding. From the crystal structure, it was observed that Tuftsin binds to the electronegative b1-domain pocket. Furthermore, the terminal arginine residue of Tuftsin appeared essential for binding. More recently, Parker et al. [108] reported on the crystal structure of VEGF-A bound to Nrp1. They found that binding occurs through both the C-terminal VEGF sequence and an exon-7 sequence. In accord with previous studies, a C-terminal arginine was essential for high-affinity binding.

The importance of a terminal arginine residue is consistent with the fact that a splice variant of VEGF lacking it (VEGF-A_165b_; SLTRKD C-terminus) fails to bind to Nrp1 [109]. Indeed, almost all VEGF-family ligands, or mimetic peptides, that bind to Nrp1 have a C-terminal arginine residue, consistent with the C-end rule ([53]; see below). This appears to apply to VEGF_121_, which was initially thought not to bind to Nrp1, but it was later reported to have affinity for this receptor [110]. The only exceptions are C7C cyclic peptides isolated from a phage library by Hong et al. [58], which had the consensus sequence –RRXR-. Interestingly, latency-associated peptide of TGF-β1 (LAP-β1) has an arginine-rich C-terminus (RHRR) and binds to Nrps, as discussed below.

## ROLE OF NRP1 IN THE IMMUNE SYSTEM

### Expression in the thymus and periphery

There are a number of areas where Nrp1 appears to contribute to immunity. It is involved in immune system development and thymocyte differentiation [3, 71-74]. It has been reported to contribute to the formation of the immune synapse between T cells and antigen-presenting cells (APCs) [111]. Thus, it may have an important role in antigen presentation, but a caveat is that effector T cells (Teff) are mostly negative for Nrp1, and only a subset of APCs (the pDCs) appears to be positive [4,75-77, 80]. In view of this, the role of Nrp1 in antigen presentation remains to be clearly defined.

### Immunoregulatory effects

Functionally, Nrp1 has been frequently linked to immune inhibition. Several studies have detailed the immunoregulatory activities of semaphorins, which can be either inhibitory or stimulatory [112]. Most of these immune effects have been attributed to SEMA classes 4, 6 and 7, which do not bind Nrps [112]. However, interactions of Nrp1 with SEMA3A can exert immune effects [113, 114]. In this case, SEMA3A forms a complex with Nrp1 (coreceptor) and plexin-A4 (signaling receptor) to activate an immunoinhibitory response. In accord with this, *Plexin-A4* knockout mice have increased antigen-induced CD4+ T-cell activation and experimental autoimmune encephalomyelitis (EAE) [114]. T cells from mice bearing a mutant Nrp1 unable to bind SEMA3, or with SEMA3A knockout, also have immunoaggressive features. SEMA3A is particularly relevant to anti-tumor immunity, because tumors frequently produce this soluble mediator, which inhibits human T-cell proliferation and cytokine production [115]. This inhibitory effect has been linked to a blockade of CD3/CD28-activated Ras/MAPK signaling [115]. This work suggests that tumors can negate anti-tumor immune responses by secreting SEMA3A, although this requires confirmation *in vivo*. A caveat is that most conventional T cells do not express Nrps, and it is difficult to envisage a general immunosuppressive mechanism based on this SEMA3A/Nrpl/PlexinA4 model without further clarification.

Alternatively, Nrp1 might exert immunoinhibitory effects by acting on Treg cells and/or enhancing responses to TGF-β1 (see below), which is a powerful immunosuppressive cytokine. As noted previously, Nrp1 is a marker of most murine Treg cells, and a subpopulation of activated human Treg cells. At least in mice, it appears to increase the length of interaction of Treg cells with antigen in the process of antigen presentation by immature DCs (iDCs), which favors the activation of Treg cells over naïve T cells [78]. Furthermore, blocking Nrp1 interferes with Treg-mediated suppression [78]. These findings suggest that Nrp1 will promote the downregulation of immune responses through increased Treg activity. This view is supported by a recent study in conditional Nrp1 knockout mice [79]. These authors found that the lack of Nrp1 on CD4+ T cells was associated with increased Th17 and decreased Treg functionality, as well as increased EAE severity. In contrast, the expression of Nrp1 by CD4+ cells was associated with suppressive activity, both *in vivo* and *in vitro*. Interestingly, Nrp1+CD4+ T cells were suppressive even when lacking the markers of classical Treg cells such as Foxp3. The suppression of Nrp1+CD4+ was inhibited by the blockade of TGF-β but not IL-10. Taken together, these findings suggest that Nrp1 expression by CD4+ cells is associated with T-cell suppressor function, and this is mediated largely by TGF-β.

### Dendritic cells

The role of Nrp1 in pDCs deserves some consideration. This small subpopulation of Nrp1+ DCs [75] is importantly involved in combating viral infections, and responds by producing large amounts of interferon α (IFN-α) [122]. The pDCs recognize viral nucleic acids through toll-like receptors (TLRs) and probably other receptors. However, beyond this conventional model, recent studies have shown they display plasticity as DCs [122]. For instance, there is evidence that pDCs can activate Treg cells [123]. Nrp1 appears to have a functional role in pDCs, because the incubation of these cells with an anti-Nrp1 antibody (BDCA-4) blocked IFN-α production induced by viral infection or nucleic acids [76]. The mechanism has not been elucidated. Intriguingly, Nrp1 is a receptor for HTLV-1 [52], and possibly other viruses, and might play an important role in viral internalization as outlined later in this review. Nrp2 may also be of importance to DCs. Indeed, Rey-Gallardo et al. [124, 125] have reported that Nrp2 promotes CCL21-driven chemotaxis and migration of mature DCs. They observed that polysialic acid, attached to Nrp2a or Nrp2b, is involved in this chemotactic process. This suggests a major biologic function for Nrp2, because DC migration to secondary lymphoid organs is a key early step in the generation of immune responses, and occurs predominantly through an interaction of the CCR7 receptor with its chemokine ligands CCL21 and CCL19.

## ROLE OF NRPS IN CANCER

### Nrp expression and prognosis

Many malignant tumor cell lines express Nrp1 and/or Nrp2, and this appears to contribute to their aggressiveness [5-11]. Clinically, as previously reviewed [5-11, 14-16], the neuropilins are frequently overexpressed in several human tumor types, including carcinomas (e.g., pancreas, prostate, breast, colon and kidney), melanoma, glioblastoma, leukemias, lymphomas and others. In general, Nrp expression correlates with more aggressive clinical tumor behavior. For instance, in breast cancer biopsies Nrp1 expression is a feature of high grade tumours, rather than low grade, and is frequently expressed by tumours of patients who died from cancer [130]. Indeed, Nrp1 or Nrp2 expression is significantly associated with poor survival in breast cancer, independent of other standard prognostic factors [131, 132].

### Does Nrp expression by tumor cells promote tumor progression?

Although Nrp expression in some types of cancers has been linked to a poor prognosis, most studies did not distinguish whether this was due to expression by the tumor vasculature or the tumor cells. Indeed, as mentioned previously, although Nrp expression by the vasculature is very common, in tumor cells it is quite variable from one tumor to another [15, 16]. Thus, it could be argued that Nrps are involved only, or mainly, in tumor angiogenesis. However, there is evidence that expression by tumor cells is relevant. For instance, Hong et al. [58] showed that Nrp1 expression was an independent predictor of poor prognosis in NSCLC. Moreover, they showed that Nrp1 knockdown in lung cancer cell lines reduced their ability to migrate, invade and form filipodia, and it also inhibited metastasis. In studies of human colon cancer cell line xenotransplantation, others demonstrated that the forced expression of Nrp2 increased tumor growth, whereas the knockdown of Nrp2 prevented tumor formation [36], or reduced tumor growth and increased apoptosis [133]. Similarly, knockdown of Nrp1 in renal cell carcinoma cells resulted in poor tumor growth [66]. In mice, the deletion of Nrp1 in normal epidermis prevented skin tumor initiation [68]. Our own studies showed that Nrp knockdown in breast cancer cell lines prevented tumor sphere formation, which is an *in vitro* assay for cancer stem cells (see below). A caveat is that these *in vivo* experimental models can only partially duplicate clinical cancer, and further studies are required to elucidate the role of Nrps in cancer progression.

### Potential Nrp-mediated actions in cancer

The precise mechanisms of action of Nrps in cancer are difficult to pinpoint, because they interact with so many cancer-associated molecules. Thus, they could be contributing to cancer cell proliferation, migration, invasion, adhesiveness and metastasis. Importantly, they appear to promote EMT and the maintenance of an immature or cancer stem cell phenotype, as discussed below. They are also expressed by various stromal cells that can interact with the tumor cells, including fibroblasts, endothelial cells and immune cells. For instance, Nrp1 was reported to bind fibronectin and activate α5β1 integrin, and to orchestrate interactions between myofibroblasts and soluble fibronectin [134]. This promoted α5β1 integrin-dependent fibronectin fibril assembly and increased matrix stiffness and tumor growth. Indeed, due to their versatility, it is likely that the neuropilins contribute to every major step in cancer biology, from tumor initiation to the generation of metastases.

Of note, Nrp2 is expressed by lymphatic endothelial cells, at least during development, and may have a special role in metastasis. It interacts with VEGF-C and its receptor VEGFR3, which are importantly involved in lymphangiogenesis [32]. Notably, Caunt et al. [135] found that an Nrp2 antibody blocked VEGF-C binding and disrupted VEGF-C-induced lymphatic endothelial cell migration. Remarkably, in tumor models, this antibody inhibited tumoral lymphangiogenesis and protected against metastasis to local lymph nodes or distant sites. Nrp2 may also increase the expression of metastatic and anti-apoptotic genes, and increase resistance to chemotherapeutic drugs, possibly through a mechanism involving β-catenin [136]. Nrp2 has also been linked to aggressive behavior in prostate cancer [137]. In this case, VEGF/Nrp2 signaling was reported to suppress IGF-1R expression, in a mechanism involving Bmi-1. Concurrent inhibition of Nrp2 and IGF-1R prevented tumor growth *in vivo*.

The cancer promoting effects of Nrps have often been attributed to an enhancement of VEGFR2 activation in response to VEGF. However, some tumours express Nrps but neither VEGFR1 nor VEGFR2 and, at least in these cases, it seems unlikely that VEGF receptors are involved. The role of semaphorins is complex, but the evidence suggests that the SEMA3s have primarily anti-cancer effects [25, 26]. As reviewed above, the Nrps interact with several GFs (VEGF, TGF-β, HGF and PDGF) that can all contribute to cancer progression. These signaling pathways interact extensively, with numerous potential outcomes (Fig. 1B). Nrps might play an important role in the regulation or coordination of these responses, especially as related to interactions between TGF-β and the other GFs.

### TGF-β signaling pathways

The three TGF-β isoforms (TGF-β1 is the most important) use the same signaling receptor and it has three major components [116]: Type I (RI, or ALK5); type II (RII); and type III (RIII, or betaglycan). RIII binds TGF-β and recruits TGF-β to RII, which then phosphorylates RI, to form a heterotetrameric serine/threonine kinase complex. In turn, RI phosphorylates Smad2 and Smad3 (receptor-associated Smads [R-Smads]), and the latter form a heteromeric complex with Smad4, which translocates to the nucleus, binds to DNA and regulates transcription. TGF-β receptors also signals through multiple noncanonical (non-Smad) pathways, including JNK/p38 MAPK, ERK1/2, PI3K/Akt, and Rho-like GTPases, and there is complex cross-talk between these pathways [138, 139]. Of particular interest, TRAF6 interactions with the TGF-β receptor complex result in the activation of TGF-β-activated kinase 1 (TAK1), which can in turn activate the JNK, p38 MAPK and NF-κB pathways [139]. Importantly, the noncanonical pathways can antagonize canonical signaling, especially by inhibiting Smad3. For instance, hyperactivation of PI3K/Akt or ERK1/2 blocks canonical signaling [138, 139, 141]. The balance between canonical and noncanonical signaling may influence tumor progression. Thus, a shift from canonical to noncanonical TGF-beta signaling may increase the likelihood of metastasis in breast cancer, as recently reviewed [142].

### Neuropilins interact with TGF-β1 and its receptors

TGF-β is quite commonly produced by tumors, and it plays an important and complex role in cancer. In early neoplastic lesions, TGF-β exerts a tumor suppressor effect, whereas at advanced stages it has a negative impact [116]. This is sometimes referred to as the TGF-β paradox, and it remains poorly understood. Indeed, in advanced disease it promotes metastasis and inhibits anti-tumor immunity [116]. A role for Nrps in modulating the response to TGF-β1 is supported by our observations that Nrp1 and Nrp2 are able to bind both active TGF-β1 and LAP-TGF-β1 (latent form) [33, 34]. Interestingly, free LAP, LAP-TGF-β1, and TGF-β1 all competed with VEGF165 for binding to Nrp1, suggesting that they bind at (or near) the same site. However, Nrp1 appears to have more than one binding site for LAP. We found that Nrp1+ T cells and cancer cells have an increased ability to capture LAP-TGF-β1. Conventional CD4+ T (lacking Nrp1) acquired strong Treg activity when coated with Nrp1-Fc and LAP-TGF-β1. Moreover, LAP-TGF-β1 was activated by Nrp1, as discussed in another section below.

Furthermore, we found that both Nrp1 and Nrp2 interact with the signaling TGF-β receptors (RI and RII) and enhances canonical Smad2/3 signaling in response to this cytokine [34]. Other authors have reported similar findings in studies of Nrp1 [35] and Nrp2 [36]. In view of these findings, we hypothesize that Nrp1 plays a key role on the membrane of cells by capturing active or latent TGF-β1, activating the latent form, and enhancing TGF-β-receptor signaling. TGF-β is involved in several aspects of regulatory T-cell biology [116]. Indeed, it promotes the survival of natural Treg (nTreg) cells, induces the differentiation of conventional T cells into induced Treg (iTreg) cells, and acts as an effector molecule for suppression. However, although Nrp1 has been linked to Treg function in mice, its role in human Treg cells is not well established because, as noted previously, only a subset of activated human Treg cells express Nrp1.

An interesting question related to cancer biology is whether TGF-β production by T cells, rather than tumor cells, is responsible for fostering tumor progression. It seems likely that both sources of TGF-β production are important. However, a recent study by Sarkar et al. [143] suggests that at least in some tumors the production of this cytokine by T cells is more relevant. In a transgenic model of mammary cancer, these authors observed that deletion of TGF-β1 from tumor cells was not protective against tumor development. In contrast, the ablation of TGF-β1 from T cells inhibited tumor growth, and prevented tumors from progressing to higher pathological grades and generating lung metastases. In the tumor environment, we speculate that Nrps could promote interactions between T cells and either tumor cells or stromal cells, leading to enhanced TGF-β1 action with its potential negative effects.

The expression of LAP on either murine or human Treg cells has been controversial, but recent studies clearly show that it is present on activated Treg cells of both species [117-120]. We identified Nrp1 as a receptor for LAP, but we observed that LAP is rapidly internalized following binding [34]. Furthermore, some T cells lacking Nrp1 express LAP, and it might not the principal receptor for membrane-attached LAP on Tregs. Indeed, other authors [117-120] have identified GARP (LRRC32) as the more likely receptor. GARP is expressed following Treg activation. It appears to anchor LAP to the membrane by covalent bonding [121]. GARP may have a functional role in Treg cells, but this remains unclear. Unlike Nrp1, GARP does not activate LAP-TGF-β1, but our results suggest the two molecules might interact in this process.

### Nrp1 and the activation of latent TGF-β on the cell membrane

TGF-β is usually secreted as a large latent complex (LLC) consisting of LAP-TGF-β (the small latent complex) that is covalently attached to a single molecule of latent TGF-β binding protein (LTBP) [116, 121]. LAP and TGF-β are not covalently bound and during activation mature TGF-β is released totally or partially, such that it can bind to the signaling TGF-β receptors. Activation is a key factor in regulating the response to TGF-β [126-129]. Cell surface molecules that capture latent TGF-beta include the RGD-binding integrins (notably the α_V_ subfamily), Nrp1 and GARP. The integrins bind the RGD motif of LAP [126-129], and some can activate TGF-β. *In vivo*, activation is thought to occur by one of two mechanisms. Some integrins, typified by α_V_β6, bind the LAP-RGD site and, concurrently, to ECM components (such as fibrillin and fibronectin) through LTBP. It appears that traction forces induce conformational changes in LAP, and release mature TGF-β. Alternatively, as typified by α_V_β8, activation is effected by MMP enzymes. However, because LAP has both an integrin-binding site (RGD) and neuropilin binding sites, we hypothesize there is a third mechanism where Nrp1 or Nrp2 contribute to the activation of latent TGF-β after it binds to an integrin. This is consistent with our recent observations that Nrp1 can activate latent TGF-β1 after it attaches to either αvβ3 integrin or another receptor (GARP) [34]. This type of interaction would be particularly relevant on the membrane of cancer cells, which frequently co-express RGD-binding integrins and neuropilins. It remains unclear how Nrp1 activates TGF-β1; however, Nrp1 and Nrp2 bear a b2 domain motif (RKFK) that we found capable of activating LAP-TGF-β1, at least in soluble peptide form [33]. In this respect, it is noteworthy that the sequence ^94^RKPK of TGF-β1 binds to LAP and, in soluble form, the RKPK peptide activates LAP-TGF-β1 [127]. This peptide is closely similar to the peptides of Nrp1 (RKFK) and thrombospondin -1 (KRFK) [33, 127] that also activate LAP-TGF-β1. It may be that these basic peptides compete with TGF-β1 for binding to LAP, and/or induce a conformational change in LAP, and this releases (partially or completely) mature TGF-β1, which can then exert its actions.

### Potential signaling activities of the Nrps

The signaling functions of the Nrps have been unclear. Interactions with the PDZ protein synectin are thought to be important, but this remains poorly understood [13]. In fact, synectin gene knockout produces a mild phenotype, compared to the lethal phenotype of Nrp1 knockout [144]. In view of this, the role of synectin requires some clarification. However, recent studies [44, 45] show that Nrp1 contributes to p130Cas phosphorylation, downstream signaling and increased cell motility in response to either VEGF, HGF or PDGF. This is dependent on the cytoplasmic segment of Nrp1, but apparently not on synectin, suggesting there is some other as yet unknown signaling machinery. Knockdown of either Nrp1 or p130Cas, or alternatively expression of either Nrp1 without its cytoplasmic domain or a non-functional p130Cas mutant, all reduced the GF-induced migration of endothelial cells and glioma cells [45]. The integrin adaptor molecule p130Cas has a large interactome. Phosphorylation of p130Cas is usually mediated by Src and FAK [145] and, interestingly, TGF-β can activate Src/FAK [141]. However, Nrp1 appears to bypass the Src/FAK pathway [44, 45]. Phospho-p130Cas is involved in the formation of a molecular complex at the cell membrane (DOCK1). This activates multiple pathways and stimulates cell proliferation, migration, survival, and invasion [145]. p130Cas contributes to transformation induced by several oncogenes (e.g., HER2, ALK, KRAS, and BRAF) [145]. In ER+ breast cancers, p130Cas is associated with disease progression and resistance to tamoxifen [146]; hence, the alternative name of breast cancer anti-estrogen resistance 1 (BCAR1).

This is particularly relevant to TGF-β action in cancer, because p130Cas has been shown to block canonical TGF-β signaling by acting on Smad3 [147, 148], whereas it increases noncanonical signaling [148]. Indeed, Wendt et al. [148] proposed that p130Cas alters the balance between canonical and noncanonical TGF-β signaling, in a way that impairs the tumor suppressor functions of TGF-β during breast cancer progression. This leads to the hypothesis that Nrps might modify the response to TGF-β in a context dependent manner (canonical vs. noncanonical), as influenced by the presence of other GFs. Thus, the tumor suppressor effects of TGF-β might depend largely on the Smad canonical pathway and dominate when the levels of other GFs are low, while the pro-metastatic effects might depend mostly on the noncanonical pathways and predominate when the levels of other GFs are high. In Figure 1B, we present a hypothetical model of how these pathways might interact.

## CANCER STEM CELLS (CSCS)

CSCs have several key features, as we have recently reviewed [70]. They express markers allowing purification, and are highly tumourigenic as compared to other tumor subsets. Other notable features include the capacity to form tumour spheres in low-adherence cultures, self-renewal, and multi-drug resistance. CSCs also express high levels of aldehyde dehydrogenase-1 (ALDH^hi^), as detected by the Aldefluor reaction. For instance, human breast CSCs have been reported to be CD44^+^, CD24^−/low^, ESA^+^, ALDH^hi^, highly tumourigenic, and responsive to TGF-β. From a clinical point of view, drug resistance is probably the most important feature. Indeed, CSCs are drug resistant and can be enriched from cancer cell lines by culture with chemotherapeutic agents (e.g., doxorubicin, mitoxantrone) [70, 149].

### Nrp1, VEGF and CSCs

VEGF appears to be important for the self-renewal, survival and tumor-forming ability of CSCs. These cells can secrete VEGF, which initiates an autocrine stimulatory loop that is dependent on both VEGFR2 and Nrp. Beck et al. [68] recently reported that VEGF affects skin tumor growth by promoting cancer stemness and CSC expansion. The deletion of Nrp1 in cutaneous CSCs blocked this ability. Similarly, Hamerlick et al. [69] found that VEGF-VEGFR2-Nrp1 signaling promotes glioblastoma CSC-like cell (CD133+) viability and tumor growth. In some glioblastoma CSCs, they observed VEGFR2-Nrp1 recycling and a pool of active VEGFR2 within a cytosolic compartment, which they postulate contributes to the resistance of these cells to anti-VEGF therapy with bevacizumab.

### NF-κB and Hedgehog (Hh) pathways in CSCs

Nrp1 and/or Nrp2 are expressed by some stem or progenitor cells [150, 151], and breast CSCs [152]. We have studied tranilast as a drug that inhibits breast CSCs by acting on the aryl hydrocarbon receptor [70, 149]. Recently, we found that tranilast markedly suppresses Nrp1 expression and NF-κB activation in breast cancer cells [152]. To examine this further, we knocked down Nrps in breast cancer cell lines with siRNA, and found this prevented tumor sphere (mammosphere) formation, which is an *in vitro* assay for breast CSCs, and abrogated constitutive NF-κB activation [152]. This is of particular interest because the NF-κB pathway contributes to mammosphere formation, and the tumorigenicity of CSCs. Our studies suggest that Nrp1 plays an important role in breast CSCs, especially as related to NF-κB activation, but the mechanisms by which it exerts these effects remain to be elucidated.

The Hh pathway is involved in key aspects of development and the maintenance of the stem cell phenotype. In this role it interacts with several other pathways regulating development, such as Wnt/β-catenin, Notch, and TGF-β. These pathways also interact in the process of epithelial-to-mesenchymal transition (EMT), which is highly important in development, wound healing and cancer. Reactivation of the Hh pathway has been linked to cancer progression, more aggressive tumor phenotypes and metastasis. The work of Hillman et al. [67] points to Nrp1 and Nrp2 as major regulatory components of the Hh pathway. These authors demonstrated that Nrps are expressed at similar times and locations as Hh during development. Moreover, Nrp1 transcription was induced by Hh signaling, and Nrp1 overexpression increased Hh target gene activation, suggesting a positive feedback circuit. With cell lines lacking Hh pathway components, they demonstrated that Nrps mediate Hh signal transduction between activated Smoothened (Smo) protein and the negative regulator Suppressor of Fused (SuFu). Similarly, Cao et al. [66] observed in a renal carcinoma model that Nrp1 knockdown resulted in a more differentiated phenotype and the inhibition of sonic Hh. They concluded that Nrp1 promotes an undifferentiated phenotype in cancer cells.

### Nrps and EMT

Nrp1 enhances signaling in three major pathways that have been linked to EMT, i.e., TGF-β, Hh and HGF/cMet. TGF-β plays a major role in EMT by regulating the expression of multiple genes and pathways, as recently reviewed by Fuxe et al. [153]. Thus, TGF-β-induced pathways interact with stem cell pathways such as Wnt, Ras, Hedgehog and Notch to produce EMT. In this process, EMT-associated transcription factors (e.g., Snail1, Zeb1/2, Twist, β-catenin) interact with Smads to form complexes that regulate the expression of epithelial and mesenchymal genes. Other pathways and miRNAs are also involved. For example, Cesi et al. [154] showed that TGF-β increases the expression of c-Myb in ER^+^ breast cancer cells by a number of mechanisms, including alterations in miRNA expression. This increase in c-Myb was required to induce the expression of EMT-associated markers, *in vitro* invasion and anchorage-independent growth. Integrins also appear to play an important role. Interestingly, Bianchi et al. [155] found that the depletion of αv-integrin or β5-integrin blocked TGF-β-induced EMT. They showed that β5-integrin adhesions contributed to the TGF-β-induced EMT and the tumorigenic potential of carcinoma cells. Because Nrps bind some integrins (Table 1), we hypothesize that Nrp/integrin complexes can form and contribute to the induction of EMT and tumor progression. Nrp interactions with PDGFs might also be relevant to EMT. Nrp1 enhances responses to PDGF-A or PDGF-B [42], and these mediators are involved in EMT [156, 157]. However, it is unknown whether Nrps interact with PDGF-C that has homology to the CUB (a1/a2) domains of Nrp1 [158] and induces VEGF-independent angiogenesis [159] and possibly EMT, or with PDGF-D that potently induces EMT [160].

Interestingly, Grandclement et al. [11, 36] identified an important function for Nrp2 in promoting EMT. In accord with our work, they showed that Nrp2 is a coreceptor for TGF-β1 and promotes Smad-dependent signaling. Indeed, Nrp2 induced EMT in a TGF-β1-dependent fashion [36]. Moreover, as mentioned previously, they showed that Nrp2 markedly enhances tumor formation in a colon cancer xenografts model. Importantly, the expression of Nrp2 was linked to constitutive canonical signaling in the TGF-β pathway. Our unpublished data suggest that Nrp1 also promotes EMT. In this respect, the studies of Mani and colleagues [161] showing that the induction of EMT in breast epithelial cells produces CSC-like cells represent a major advance. This demonstrates an inducible program that promotes cancer progression, and that might be amenable to therapy. Taken together, these findings suggest that Nrps contribute to EMT, which has been associated with a CSC phenotype and aggressive tumor behavior.

## NRP1 AND NRP2 AS TARGETS FOR CANCER THERAPY

There is great interest in targeting Nrps for cancer therapy, and various approaches have been advocated. Most of these therapies have been reviewed by others in recent years [5-11]. One of the earliest approaches involved the administration of soluble Nrp1 variants to act as a VEGF trap, and this showed some anti-cancer therapeutic benefits. Based on current knowledge, it is likely that other growth factors were also blocked, but this has not been examined. Other approaches include Nrp blockade with antibodies or peptides, or knockdown with siRNA or shRNA. For example, peptides can compete with VEGF for binding to Nrp1 [53-62]. In some studies, Nrp1-binding peptides or knockdown of Nrp1 by siRNA inhibited cancer cell growth and increased the sensitivity of the cells to chemotherapeutic agents (e.g., 5-FU, paclitaxel, and cisplatin) [61]. Of note, Nrp1 may be a useful target for therapy in glioblastoma [162], melanoma [163] and some forms of leukemia [62].

The b1-domain binding site for the C-terminal motif of VEGF has been very well defined by crystallographic studies. This facilitates the design of small drugs that fit into this site, to block VEGF binding. These drugs would likely also block other ligands that are dependent on that binding site. Jarvis et al. [164] recently reported on the molecular design of such a small molecule ligand (EG00229). This drug inhibited VEGF-A binding to NRP1, and reduced the viability of A549 lung carcinoma cells. Remarkably, it also increased the potency of the anti-cancer cytotoxic agents paclitaxel and 5-fluorouracil. The clinical availability of small drugs of this kind, especially if orally active, would represent a major advance in the area of Nrp-targeted therapy.

It is of particular interest that combined anti-VEGF and anti-Nrp1 therapy with monoclonal antibodies was synergistic in mouse models of cancer [104]. Since the SEMA3s appear to exert primarily anti-cancer effects whereas the VEGFs tend to promote cancer, it could be beneficial to block only VEGF binding. This might be possible with specific antibodies or peptides. Genentech has developed antibodies against the SEMA3 binding “a” region (anti-Nrp1^A^) or VEGF binding “b” region (anti-Nrp1^B^) [104]. However, these two antibodies had similar effects on reducing angiogenesis and vascular remodeling [104]. The reason is unclear, although we speculate that both antibodies induce endocytosis of Nrp1, and hence have a similar therapeutic activity. Interestingly, blocking Nrp1 had only a minor effect on VEGFR2 signaling showing that some Nrp and VEGFR2 functions can be dissociated, as recently reviewed by Zachary [13].

Nrp2 has been more closely linked to metastasis than Nrp1 and, therefore, represents a key target. Indeed, an antibody binding the VEGF bindings of Nrp2 (anti-NRP2^B^) has been developed, and found to inhibit tumour lymphatic development and prevent metastasis [135]. Since Nrp1 and Nrp2 have similar but not completely overlapping roles in angiogenesis and cancer, it might be desirable to therapeutically block both. These approaches involving Nrp blockade clearly have merit, but there are also some potential drawbacks. Nrp1 and/or Nrp2 are expressed by endothelial cells throughout the vascular system (arteries, veins and lymphatics), as well as several other cell types, and therapies targeting these cells may have adverse effects. There is currently very little information on this subject, but a phase I clinical trial (Genentech, Inc.) with the human anti-NRP1 antibody MNRP1685A resulted in transient platelet depletion [165]. Furthermore, analysis of the safety profile of this antibody when combined with bevacizumab (anti-VEGF antibody), with or without paclitaxel, revealed a high incidence of proteinuria [166]. Thus, although anti-Nrp1 therapy may not be markedly toxic on its own, it may produce important adverse effects when combined with some anti-cancer agents.

### Nrp1 and the C-end rule

A recent finding is that peptides that bind to Nrp1 are quickly internalized. Since cancer cells frequently express Nrp1, it could be a target permitting internalization of many drugs into these cells. This would be particularly useful in the case of large drugs that cannot cross the membrane. Teesalu et al. [53] screened a phage peptide library with the goal of identifying cell-penetrating peptides (CPPs). They observed that many CPPs in their library bound to Nrp1 and had a C-terminal consensus ^R^/_K_XX^R^/_K_ motif, preferentially with a C-terminal arginine (R) although occasionally lysine (K). These peptides appear to bind to the electronegative pocket of the b1 domain of Nrp1, as noted previously for the C-terminal motif of VEGF. These authors denoted this binding pattern the C-end rule (CendR). Of importance for cancer therapy, Sugahara et al. [54, 55] described tumor-homing cyclic peptides designated iRGD (CRGD^K^/_R_GP^D^/_E_C), which attached to RGD-binding integrins through their RGD motif. They reported that these peptides were cleaved on the membrane of tumor cells by a furin-like protease, and this exposed a CendR motif (RGDK/R) that could bind to Nrp1. Importantly, attachment of this peptide to Nrp1 resulted in peptide/Nrp1 internalization, along with peptide-linked cargo. The precise mechanism of internalization has not been elucidated. Furthermore, the iRGD peptides induced vascular leakage and allowed extensive tissue penetration of the peptide and attached cargo, especially in tumors. Drugs coinjected with a CendR peptide also showed increased penetration into tumors, presumably due to increased vascular permeability [55]. Subsequent studies have shown that an RGD motif is not always required for the homing of CendR peptides to tumors, and that Nrp2 can also internalize peptides through the CendR pathway [167]. These findings are interesting, but the importance of CendR peptides in cancer therapy remains to be established, especially as to their specificity and safety.

It is also interesting to note that many proteins have a CendR motif, which is either constantly exposed (e.g., VEGF and LAP-β1), or exposed following enzymatic cleavage on the cell membrane (e.g., viral capsid proteins) [53-57]. For instance, we observed that LAP-β1 (RHRR CendR motif) is rapidly internalized into tumor cells after it binds to Nrp1 [33]. Due to this cell-penetrating ability, CendR motifs are likely of biological importance in several situations, such as internalization of bacterial toxins [53] and infection with some viruses such as HTLV-1 [168]. Some animal toxins also have a CendR motif, such as the cell-penetrating Imperatoxin A of scorpions [169], and this might be relevant to their toxicity but it remains to be examined.

## CONCLUSIONS

The Nrps are versatile proteins interacting with numerous ligands and involved in cardiovascular and nervous system embryonic development, as well as post-natal angiogenesis, immunity and cancer. Their interactions with SEMA3 and VEGF soluble ligands and their receptors have been extensively studied. Despite this, the actual molecular mechanisms of action of Nrps remain poorly understood, especially as related to signaling. The recent identification of several other ligands such as TGF-β, HGF and PDGF has raised even more questions. Interestingly, a general pattern has been that Nrps interact with both the soluble ligands and their classic signaling receptors. Furthermore, these ligands are involved in orchestrating angiogenesis in various functions, including VEGFs, TGF-β, HGF and PDGF. The Nrps are not absolutely required for signaling in these pathways, but they usually boost or alter the response. We can hypothesize that Nrps play some role in capturing the ligands, regulating GF receptor expression, endocytosis and recycling, or possibly signaling independently. In the case of the SEMA3s, except for SEMA3E, Nrps are essential for activating the plexin receptors, and these SEMA3/Nrp/Plexin interactions frequently antagonize VEGF-induced stimulation, especially as related to angiogenesis and cancer. Although SEMA3s were not a major focus of this review, their importance in tumor biology is considerable and their action is complex, as detailed in reviews by other authors [7, 25, 26, 170-174]. In the immune system, the Nrps are expressed mainly by DCs and Tregs, and have been linked chiefly to inhibitory effects. This may depend on Nrp-mediated T-cell/DC interactions or the capture and activation of latent TGF-β, and these mechanisms are not mutually exclusive. Inhibition may also be mediated by SEMA3A, as suggested by some studies. At any rate, conditional Nrp1 knockout in murine T cells results in poor Treg function and increased autoimmunity. This suppressive role of Nrp1 is particularly relevant to cancer, where it could block anti-tumor immunity.

In cancer, Nrp expression has frequently been linked to a poor prognosis. This may be due to increased tumor angiogenesis, but it is clear that Nrps mediate other tumor promoting effects. For instance, Nrp1 promotes TGF-β, NF-κB and Hedgehog signaling, EMT and CSC survival. Nrp2 expression by tumor cells and lymphatic endothelial cells has been linked to metastasis. Importantly, Nrps appear to activate pathways that protect tumor cells against apoptosis and cytotoxic anti-cancer drugs. The identification of CendR peptides that penetrate the cell membrane by binding to Nrp1 is of major therapeutic interest. These peptides (or larger proteins with a CendR C-terminal motif) have considerable cell- and tissue-penetrating ability. Thus, they can be coupled to drugs (cargo), especially large drugs, which do not normally penetrate the cell membrane. All these features point to Nrps as important targets for cancer therapy. This can be accomplished by several methods, as reviewed above, but almost all these studies have been preclinical, and much more research is needed to translate these findings into clinically applicable therapies.
